# Relationship between medication burden and medication experience in stable patients with schizophrenia: the mediating effect of medication belief

**DOI:** 10.1186/s12912-024-01882-4

**Published:** 2024-03-22

**Authors:** Yujing Sun, Hong Yu, Zhengjun Wang, Jing Zhang, Yuqiu Zhou, Wenming Cui, Wenlong Jiang

**Affiliations:** 1 Department of Nursing, Harbin Medical University Daqing Campus, Daqing, China; 2 The Fourth Affiliated Hospital of Harbin Medical University, Harbin, China; 3 Daqing Third Hospital, Daqing, China

**Keywords:** Schizophrenia, Medication burden, Medication experience, Medication belief

## Abstract

**Background:**

Individuals with schizophrenia require prolonged antipsychotic medication treatment. But more than 50% of individuals with schizophrenia experience adverse medication experiences during their antipsychotic treatments. Such individuals often adjust or discontinue medication, leading to disease relapse and impaired social functioning. Psychiatric nurses should pay close attention to the medication experiences of individuals with schizophrenia. This research explore the relationship between medication burden and medication experience, as well as the mediating effect of medication belief in stable patients with schizophrenia.

**Methods:**

A convenience sample of hospitalized stable patients with schizophrenia were selected from Daqing Third Hospital and Baiyupao Hospital from September 2023 to December 2023. A survey was conducted with them using a questionnaire consisting of general information questionnaire, The Subjective Well-being Under Neuroleptic Treatment Scale(SWN), The Living with Medicines Questionnaire(LMQ), Beliefs about Medicines Questionnaire-Specific (BMQ-Specific). Pearson correlation analysis was used to explore the correlation between LMQ, BMQ-Specific and SWN scores, and multiple linear regression analysis was used to explore the influencing factors of medication experience in patients with schizophrenia. AMOS 24.0 was used to construct the structural equation modeling(SEM), and the mediation effect of the SEM was tested using Bootstrap method.

**Results:**

According to the sample size calculation requirements of structural equation model, a total of 300 samples were required in this study, and 400 effective questionnaires were actually collected in this study, which met the sample size requirements for constructing structural equation models. Bootstrap test showed that the mediation effect was significant. The total effect of medication burden on medication experience was significant (Z=-12.146, 95%CI (-0.577, -0.417), *P* < 0.001). The indirect effect of medication burden on medication experience, that is, the mediating effect of medication belief was significant (Z=-4.839, 95%CI (-0.217, -0.096), *P* < 0.001). The direct effect of medication burden on medication experience was significant (Z=-7.565, 95%CI (-0.437, -0.257), *P* < 0.001). This model belongs to partial mediation model.

**Conclusions:**

Psychiatric nurses can enhance the patients’ medication experience by reducing medication burden and strengthening medication beliefs. Therefore, the results also provide theoretical references and decision-making foundations for psychiatric nursing professionals to develop appropriate management strategies for individuals with schizophrenia.

## Background

Schizophrenia is a severe mental disorder affecting approximately 20 million people worldwide [1]. By 2020, China had recorded 6.43 million individuals with severe mental disorders; 4.58 million people were diagnosed with schizophrenia, accounting for 71.28% of all cases [2]. This illness shortens both overall survival (OS) and quality-adjusted life years (QALY) by 20.6 and 18.4 years, respectively [3]. Schizophrenia is expected to be the most common condition among various psychiatric disorders, with an estimated escalation of the global expenditure of mental disorders to $6 trillion by 2030 [4]. Therefore, psychiatric nurses cannot afford to overlook the essential aspect of care management that focuses on improving the disease outcomes of individuals with schizophrenia.

The term “medication experience” describes the patient’s cognitive and emotional perception regarding pharmacological therapy’s benefits and limitations [5]. Currently, very few self-reported studies have analyzed the medication experience of individuals undergoing treatment for schizophrenia. Most of the studies have primarily predicted “treatment non-adherence” rather than exploring the medication experience itself [6, 7]. Individuals with schizophrenia require prolonged antipsychotic medication treatment. A study indicated that > 50% of individuals with schizophrenia experience adverse medication experiences during their antipsychotic treatments [8]. Consequently, such individuals often adjust or discontinue medication, leading to disease relapse and impaired social functioning [9, 10]. Therefore, psychiatric nurses should pay close attention to the medication experiences of individuals with schizophrenia. Hence, improving medication experiences can enhance medication adherence, reduce relapse and readmission rates, and improve the overall quality of life and prognosis of such individuals.

Medication burden refers to the stress that patients experience during acquiring, planning, and organizing medication, adhering to medication regimens, monitoring progress, and managing medication-related adverse reactions. This encompasses the burden associated with daily medications, drug characteristics, adverse reactions, as well as medication-related social burden [5, 11, 12]. Some individuals with schizophrenia experience noticeable medication-related adverse reactions like weight gain, sleep disturbances, and impaired attention that significantly impact their medication experience [13]. Moreover, individuals with schizophrenia bear the highest medication-related medical burden among all psychiatric disorders [14]. A study indicated that the yearly economic burden for individuals with schizophrenia ranges from $2004 to $94,229 [15]. Individuals with schizophrenia experience multifaceted medication burdens during pharmacological treatment, which influence their medication experiences [5]. Therefore, we proposed as our first hypothesis that medication burden can directly influence medication experience.

Medication belief describes an individual’s cognitive assessment of the benefits and drawbacks associated with medication adherence, encompassing both the belief in the necessity of medication and concerns about its safety [16]. It represents a modifiable cognitive factor. Research indicates that a stronger conviction regarding the medication’s necessity is associated with an enhanced perception of medication benefits during the adherence process, thereby leading to positive medication experiences [17]. Conversely, higher medication concerns can increase the fear of adverse reactions, resulting in poor adherence and negative medication experiences. This might contribute to low medication adherence behaviors [17]. Therefore, we proposed our second hypothesis that medication beliefs have a direct predictive effect on medication experiences.

Medication burden is a key factor influencing patients’ medication beliefs, and a negative correlation has been observed between medication beliefs and burden [18]. Positive medication beliefs help patients cope with negative emotions during the medication process, maintain a positive attitude, and reduce medication burden[19]. Thus, patients’ medication burden and beliefs can significantly influence patients’ medication experiences [5, 20, 21]. Therefore, our third hypothesis posits that medication beliefs function as an intermediate variable between medication burden and experiences.

Although the impact of medication burden and beliefs on medication experience has been highlighted before, the inherent connections between these variables have been explored very little. The structural equation model can reveal the relationship between variables, quantify the size of the mediation effect, help us understand the mechanism of action between variables, and improve the accuracy and interpret ability of the research. Given this background and our hypotheses, this study aimed to explore the relationships among medication burden, medication beliefs, and medication experience in individuals with schizophrenia by constructing a structural equation model. We also aspired to elucidate the underlying mechanisms to establish a theoretical basis for psychiatric nurses to develop relevant intervention measures aimed at improving patients’ adverse medication experiences.

## Materials and methods

### Design

Using a convenience sampling approach, we selected two psychiatric specialty hospitals in Daqing and Harbin, Heilongjiang Province, China as study hospitals from September 2023 to December 2023. Hospitalized stable patients with schizophrenia meeting the inclusion criteria were chosen as the study subjects. The average value of 40.5% [(27%+54%) /2 = 40.5%] was considered for sample size calculation, based on the estimated range of adverse medication experience occurrence rates in patients with schizophrenia, with low and higher limits of 27% and 54%, respectively [8]. The sample size (n) was calculated by using the formula:

$$\mathbf{n}=\mathbf{u}^{2}_{\mathbf{\alpha}/2}\mathbf{\pi}(1-\mathbf{\pi})/\mathbf{\delta}^{2},$$

where **n** is the estimated sample size, **π** is the population rate, **u**_**α/2**_ is the value corresponding to the 95% confidence interval under the normal distribution curve (assuming a significance level **α** = 0.05, **u**_**α/2**_ = 1.96, and **δ** is the allowable error. We set the maximum allowable error as 5%. The calculated sample size was approximately n = u^2^_α/2_π(1-π)/*δ*^2^ = 1.96^2^ × 0.405(1–0.405)/0.05^2^≈370. Thus, our final sample size was 400 after considering potential influences such as dropouts, invalid questionnaires, and the model’s stability. According to the sample size calculation requirements of structural equation model, that is, each observed variable requires at least 10 to 20 samples. In this study, there are 15 observed variables, and the sample size obtained by calculation is 300 cases. The sample size of 400 cases in this study meets the requirement of constructing structural equation model.

### Participants

Stable patients with schizophrenia means that patients leave the acute stage of the disease, their self-awareness is basically restored, and hallucinations and delusions are basically disappeared: and the symptoms are mainly negative symptoms and cognitive impairment [22]. Diagnostic interviews in this study were conducted independently by two deputy chief physician using the International Classification of Diseases-10 diagnosis criteria. Inclusion criteria: ① patients who met the International Classification of Diseases-10 diagnosis criteria for schizophrenia; ② patients who are 18–60 years old; ③ patients who achieved the stable period through oral antipsychotic drug treatment, as judged by the following criteria: delusion, hallucinatory behaviors, exaggeration, and suspicion/victimization items in the positive and negative symptom scale (PANSS), abnormal thought content scores of ≤ 5 in the general psychopathology scale, and PANSS conceptual disorganization scores of ≤ 4, if all the aforementioned criteria were met, the patient was considered to be in a stable period of schizophrenia [23]; ④ patients with partial insight (PANSS G12 score < 4) [24]; ⑤ patients possessing communication and comprehension abilities, and those who could complete the given assessment; and ⑥ patients who voluntarily participated in this study. Exclusion criteria: patients with a coexisting history of mental developmental delay and other mental disorders or those who were accompanied by severe physical illnesses. Ethical approval was obtained from the Ethics Committee of Harbin Medical University, and it conformed to the ethical guidelines of the Helsinki Declaration. All enrolled patients signed informed consent.

We employed a face-to-face data collection method. Before the survey, investigators underwent standardized training that covered topics like the survey’s purpose, communication language during the survey, potential issues about the survey and their resolutions, on-site verification of survey questionnaires, etc. The training duration was approximately 20 min. Before completing the questionnaires, all participants provided informed consent after being fully informed about the questionnaire and relevant precautions. Patients proficient in both spoken and written Chinese independently completed the questionnaires. Researchers used a question-and-answer format to complete the questionnaires for patients facing difficulties in reading and writing. The estimated time for questionnaire completion was approximately 15 min. The completeness and standardization of the questionnaires were cross-checked on the spot after survey completion. Questionnaires with no missing items and logical consistency were considered valid.

### Measures

The general information questionnaire designed by the research team, includes gender, age, education level, marital status, number of recurrence, duration of disease, age of onset, type of medication, duration of medication. The second edition of the guidelines for the prevention and treatment of schizophrenia in China pointed out that patients with schizophrenia who recurrences more than 3 times within 5 years should be treated for a long time, and no more than 3 recurrences within 5 years can be treated for a short time. Therefore, this study divided the number of recurrences categorized as ≤ 3 and > 3. Patients with schizophrenia whose duration of disease is less than 3 years are defined as first-episode patients [25]. According to this, the duration of disease and the duration of medication were categorized as ≤ 3 and > 3.

The Subjective Well-being Under Neuroleptic Treatment Scale (SWN) scale was translated into Chinese by Guo Jiyuan et al. at Beijing Minkang Hospital in 2003. After clinical testing, Cronbach’s α coefficient of 0.839 was obtained, indicating good reliability [26, 27]. It comprises 20 items, organized into five key factors: mental functioning, self-control, emotional regulation, physical functioning, and social integration. Utilizing a 6-point Likert scale, 1 = not at all, 2 = a little, 3 = somewhat, 4 = noticeable, 5 = much, 6 = very much, it assesses the patient’s subjective experiences over the past week, with total scores ranging from 20 to 120 points. A total score < 80 indicates the occurrence of adverse medication experiences, while higher scores suggest improved patient comfort. Hence, we used the SWN scale to evaluate patients medication experiences. The scale’s Cronbach’s α coefficient in this study was measured to be 0.990. AMOS 24.0 software was used for confirmatory factor analysis of SWN scale to test the degree of fitting between theoretical hypothesis and actual sample data. *X*^2^/*df* = 2.381, GFI = 0.990, NFI = 0.972, IFI = 0.984, TLI = 0.979, CFI = 0.984, RMSEA = 0.059. The results show that the fitting index reaches or approaches the recommended value, the model fits well and has good structural validity.

The Living with Medicines Questionnaire (LMQ) was developed by British scholars, including KRSKA in 2013 [28] and translated into Chinese by Wang et al. [29]. Demonstrating good reliability and validity, this questionnaire comprises 39 items organized into eight dimensions, including medication attitude (7 items), doctor-patient relationship (5 items), practical difficulties (6 items), medication effects (5 items), side effects (4 items), economic burden (3 items), interferences with daily life (6 items), and medication behavior (3 items). Utilizing a 5-point Likert scale, higher LMQ scores indicate a higher medication burden. Hence, the LMQ questionnaire was employed to assess the medication burden in individuals with schizophrenia. The scale’s Cronbach’s α coefficient in this study was measured to be 0.993. AMOS 24.0 software was used for confirmatory factor analysis of LMQ scale to test the degree of fitting between theoretical hypothesis and actual sample data. *X*^2^/*df* = 2.402, GFI = 0.834, NFI = 0.946, IFI = 0.968, TLI = 0.964, CFI = 0.968, RMSEA = 0.059. The results show that the fitting index reaches or approaches the recommended value, the model fits well and has good structural validity.

Developed by Horne et al. [30], the Beliefs about Medicines Questionnaire-Specific (BMQ-Specific) tool consists of two 5-item dimensions: Specific Necessity and Specific Concerns, totaling 10 items. On a 5-point Likert scale, from “strongly disagree” to “strongly agree”, each item is scored from 1 to 5. Each dimension has a score range from 5 to 25, with higher scores indicating enhanced beliefs in the corresponding dimension. The total medication belief score is calculated as the score of Specific Necessity minus the score of Specific Concerns. The scale’s Cronbach’s α coefficient is 0.77; Cronbach’s α coefficients for the Specific Necessity and Specific Concerns dimensions range from 0.55 to 0.86 and 0.63 to 0.80, respectively. These values indicate good reliability and satisfactory internal consistency. Hence, the BMQ-Specific questionnaire was utilized to assess medication beliefs in individuals with schizophrenia. The scale’s Cronbach’s α coefficient in this study was measured to be 0.950. AMOS 24.0 software was used for confirmatory factor analysis of BMQ-Specific scale to test the degree of fitting between theoretical hypothesis and actual sample data. *X*^2^/*df* = 1.224, GFI = 0.980, NFI = 0.990, IFI = 0.998, TLI = 0.998, CFI = 0.998, RMSEA = 0.024. The results show that the fitting index reaches or approaches the recommended value, the model fits well and has good structural validity.

### Statistical analysis

Data analysis was conducted using SPSS 25.0 software. Normally distributed variables were presented as mean ± standard deviation. Independent sample t-tests and one-way analysis of variance (ANOVA) were used for inter-group and multiple-group comparisons, respectively. Pearson’s correlation analysis was used for correlation analysis. Multiple linear regression analysis was utilized to analyze the factors influencing medication experience in individuals with schizophrenia. All *p* < 0.05 values were considered statistically significant.

Structural equation modeling (SEM) was established using AMOS 24.0 software. The maximum likelihood (ML) method was used to estimate the hypothesized model. The following fit indices evaluated the overall model fit, including goodness of fit index (GFI), adjusted goodness of fit index (AGFI), normed fit index (NFI), Tucker-Lewis index (TLI), comparative fit index (CFI), and incremental fit index (IFI). Scores > 0.90 indicate a reasonable fit, with higher scores indicating a better fit. The root mean square error of approximation (RMSEA) was used for evaluating the model fit, with values < 0.05 and < 0.08 indicating good and reasonable fit, respectively. The bootstrap method helped in mediation analysis, with 5,000 bootstrap samples. A significant mediating effect was seen if the 95% confidence interval (CI) did not include 0. The partial mediation model requires that the independent variable can directly and indirectly influence the dependent variable through a mediator [31].

## Results

### Demographic and medical characteristics of the sample

A total of 410 questionnaires were sent out in this study, and 400 were effectively collected, with an effective recovery rate of 97.56%. The incidence of adverse medication experiences in this study was 43.75%(175/400). Duration of disease with a mean score of (10.13 ± 7.50) years. We used SWN scale to evaluate patients’ medication experience, which was the dependent variable of this study. Therefore, we only compared the relationship between the SWN scale and other variables. The SWN scores of patients with different number of recurrence, duration of disease and duration of medication had statistical significance (*P* < 0.05), as shown in Table 1.

**Table 1:** Comparison of SWN scores in patients with different general conditions

Variables	N(%)	SWN score	F(t)	P
Gender			−1.345^(1)^	0.180
Male	202(50.50%)	80.18 ± 30.777		
Female	198(49.50%)	84.27 ± 30.050		
Age			1.492^(2)^	0.226
<30	44(11.00%)	82.45 ± 30.765		
30 ~ 50	307(76.75%)	81.07 ± 30.627		
>50	49(12.25%)	89.14 ± 28.679		
Education level			1.033^(2)^	0.378
Primary school	90(22.50%)	83.84 ± 30.604		
Junior middle school	212(53.00%)	81.27 ± 30.426		
High school	55(13.75%)	87.13 ± 29.785		
Junior college and above	43(10.75%)	77.12 ± 31.050		
Marital status			0.811^(2)^	0.488
Unmarried	176(44.00%)	80.77 ± 30.443		
Married	137(34.25%)	81.22 ± 30.725		
Divorced	84(21.00%)	86.58 ± 29.996		
Widowed	3(0.75%)	89.33 ± 34.962		
Number of recurrence			2.529^(1)^	0.012
≤ 3 times	208(52.00%)	85.89 ± 29.490		
>3 times	192(48.00%)	78.22 ± 31.043		
Duration of disease			−2.426^(1)^	0.016
≤ 3 years	133(33.25%)	76.93 ± 31.116		
>3 years	267(66.75%)	84.84 ± 29.825		
Age of onset			−0.621^(1)^	0.535
≤ 30 years old	272(68.00%)	81.56 ± 30.300		
>30 years old	128(32.00%)	83.59 ± 30.842		
Type of medication			0.597^(1)^	0.551
≤ 3 type of medication	194(48.50%)	83.14 ± 30.645		
>3 type of medication	206(51.50%)	81.33 ± 30.314		
Duration of medication			2.053^(1)^	0.041
≤ 3 years	159(39.75%)	86.02 ± 29.816		
>3 years	241(60.25%)	79.69 ± 30.664		

(1) is the t test; (2) is the one-way analysis of variance (ANOVA)

### Multiple linear regression analysis of influencing factors for medication experience in stable patients with schizophrenia

Multiple linear regression analysis was conducted with the medication experience of patients with schizophrenia as the dependent variable and the number of recurrence, duration of disease, duration of medication, medication burden and medication belief as the independent variables. The dependent variable of this study is a continuous variable. The results showed that medication burden and medication belief were the influencing factors of medication experience of schizophrenia patients (*P* < 0.001), as shown in Table 2.

**Table 2:** Multiple linear regression analysis results of influence factors for medication experience in patients with schizophrenia

Variables	B	SE	β	t	P
Number of recurrence	−2.061	2.622	−0.034	−0.786	0.432
Duration of disease	2.895	2.740	0.045	1.056	0.291
Duration of medication	−2.754	2.654	−0.044	−1.038	0.300
Medication Burden	−0.296	0.033	−0.405	−9.113	<0.001
Medication Belief	0.834	0.112	0.326	7.459	<0.001

*Abbreviations*: *SE*, standard error; *B*, unstandardized coefficient; *β*, standardized coefficient

### Pearson’s correlations among medication burden, medication belief and medication experience in stable patients with schizophrenia

Pearson’s correlation analysis showed that medication burden was negatively correlated with medication experience (*r*=-0.502, *P* < 0.01), medication burden was negatively correlated with medication belief (*r*=-0.345, *P* < 0.01), and medication belief was positively correlated with medication experience (*r* = 0.462, *P* < 0.01), specific necessity was positively correlated with medication experience (*r* = 0.463, *P* < 0.01) and specific concerns was negatively correlated with medication experience (*r*=-0.369, *P* < 0.01). The reason for this result may be related to the scoring rules of the scale. The total medication belief score is calculated as the score of Specific Necessity minus the score of Specific Concerns. The scores and correlation analysis results of each scale and its sub-dimensions are shown in Table 3.

**Table 3:** Correlation analysis results of medication burden, medication belief and medication experience in patients with schizophrenia

Variables	Mean	SD	1	2	3	4	5	6	7	8	9	10	11	12	13	14	15	16	17	18
**1**	82.21	30.450	1																	
**2**	14.78	6.310	0.973**	1																
**3**	18.03	6.140	0.982**	0.948**	1															
**4**	17.21	6.522	0.968**	0.923**	0.946**	1														
**5**	17.45	6.197	0.966**	0.924**	0.939**	0.913**	1													
**6**	14.74	6.184	0.967**	0.927**	0.938**	0.917**	0.916**	1												
**7**	93.57	41.636	-0.502**	-0.480**	-0.477**	-0.523**	-0.495**	-0.463**	1											
**8**	18.15	8.409	-0.493**	-0.468**	-0.469**	-0.515**	-0.486**	-0.455**	0.986**	1										
**9**	11.94	6.693	-0.497**	-0.473**	-0.472**	-0.512**	-0.495**	-0.460**	0.977**	0.965**	1									
**10**	11.14	5.242	-0.421**	-0.403**	-0.397**	-0.446**	-0.414**	-0.381**	0.967**	0.950**	0.940**	1								
**11**	11.03	5.069	-0.482**	-0.458**	-0.458**	-0.506**	-0.473**	-0.445**	0.971**	0.958**	0.944**	0.934**	1							
**12**	14.81	6.295	-0.479**	-0.456**	-0.450**	-0.504**	-0.470**	-0.446**	0.967**	0.953**	0.937**	0.943**	0.940**	1						
**13**	10.19	4.775	-0.511**	-0.489**	-0.486**	-0.528**	-0.506**	-0.472**	0.979**	0.966**	0.955**	0.945**	0.950**	0.944**	1					
**14**	9.57	4.399	-0.419**	-0.408**	-0.404**	-0.426**	-0.409**	-0.385**	0.704**	0.641**	0.646**	0.615**	0.631**	0.599**	0.643**	1				
**15**	6.76	3.110	-0.481**	-0.467**	-0.458**	-0.496**	-0.472**	-0.442**	0.950**	0.934**	0.922**	0.915**	0.923**	0.915**	0.935**	0.618**	1			
**16**	2.03	11.882	0.462**	0.438**	0.427**	0.467**	0.476**	0.433**	-0.345**	-0.332**	0-0.317**	-0.362**	-0.341**	-0.349**	-0.350**	-0.191**	-0.359**	1		
**17**	16.18	6.631	0.463**	0.446**	0.426**	0.464**	0.475**	0.439**	-0.347**	-0.336**	0-0.325**	-0.364**	-0.332**	-0.339**	-0.346**	-0.216**	-0.357**	0.903**	1	
**18**	14.15	6.541	-0.369**	-0.344**	-0.344**	-0.378**	-0.384**	-0.342**	0.275**	0.262**	0.246**	0.289**	0.282**	0.290**	0.286**	0.127*	0.290**	-0.901**	-0.627**	1

***p < 0.01

*Note*: SD, Standard deviation; 1 = SWN score; 2 = mental functioning; 3 = self-control; 4 = emotional regulation; 5 = physical functioning; 6 = social integration; 7 = LMQ score; 8 = medication attitude; 9 = practical difficulties; 10 = doctor-patient relationship; 11 = medication effects; 12 = interferences with daily life; 13 = side effects; 14 = medication behavior; 15 = economic burden; 16 = BMQ-Specific score; 17 = Specific Necessity;18 = Specific Concerns

### Structural equation modeling with latent variable

**Fig. 1:**
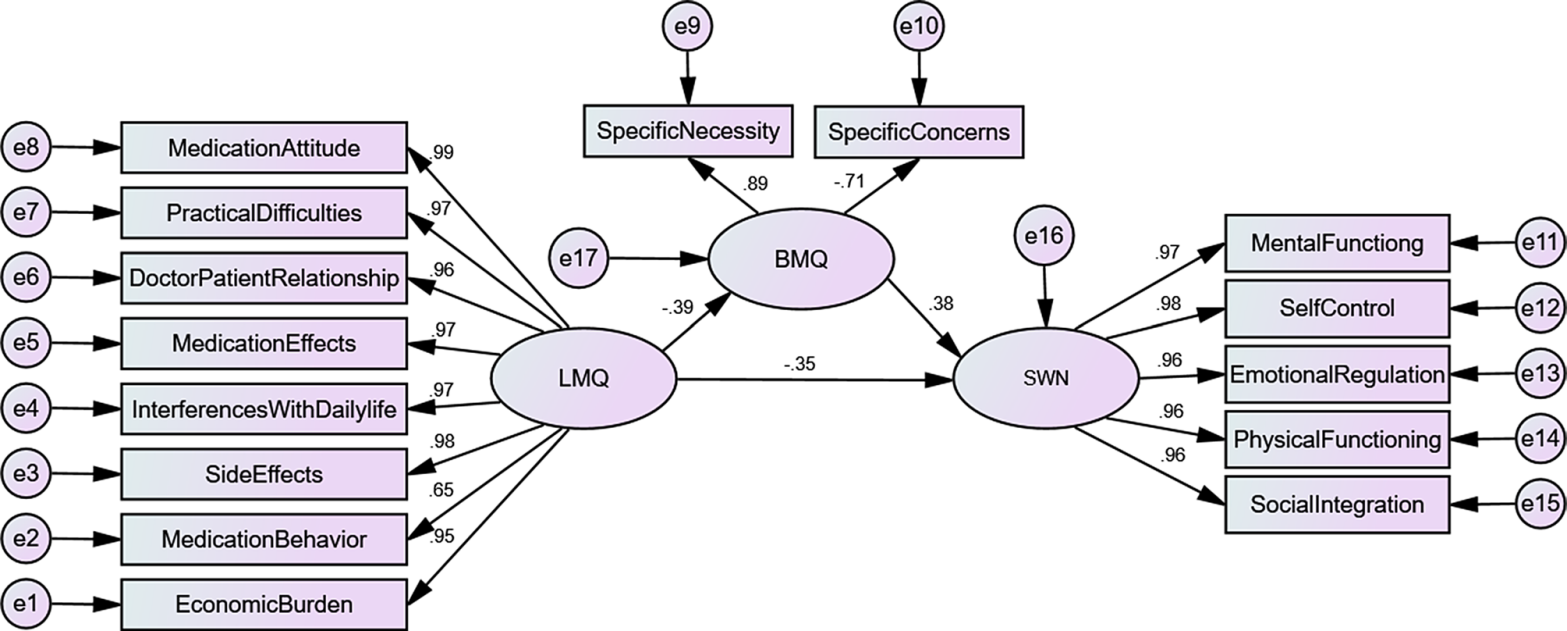
The mediation model of medication belief between medication burden and medication experience in patients with schizophrenia

The structure of the hypothesis model is estimated and tested by maximum likelihood method. Finally, a structural equation model with medication burden as an independent variable and medication belief as an intermediary variable was formed, which jointly acted on medication experience (Fig. 1). The test results show that the model has a good fit. *X*^2^/*df* = 2.332, GFI = 0.938, AGFI = 0.915, NFI = 0.981, IFI = 0.989, TLI = 0.987, CFI = 0. 989. RMSEA = 0.058, the fit of the model is reasonable.

### The mediation analysis of medication belief in the relationship between medication burden and medication experience in stable patients with schizophrenia

The significance of the mediation effect was tested using the Bootstrap program. As shown in Table 4 and 95%CI of each mediation path did not include 0, and the mediation effect was significant. The total effect of medication burden on medication experience was significant (Z=-12.146, 95%CI (-0.577, -0.417), *P* < 0.001). The indirect effect of medication burden on medication experience, that is, the mediating effect of medication belief was significant (Z=-4.839, 95%CI (-0.217, -0.096), *P* < 0.001). The direct effect of medication burden on medication experience was significant (Z=-7.565, 95%CI (-0.437, -0.257), *P* < 0.001). This model belongs to partial mediation model

**Table 4:** Bootstrap analysis of medication burden and medication belief on medication experience in patients with schizophrenia

Pathway	β	95%CI	SE	Z	P
Total effect					
Medication Burden→Medication Experience	−0.498	(−0.577,−0.417)	0.041	−12.146	<0.001
Indirect effect					
Medication Burden→Medication Belief→Medication Experience	−0.150	(−0.217,−0.096)	0.031	−4.839	<0.001
Direct effect					
Medication Burden→Medication Experience	−0.348	(−0.437,−0.257)	0.046	−7.565	<0.001

## Discussion

Our results indicate that medication burden can directly predict medication experience. Specifically, higher medication burden levels are associated with poorer medication experiences, consistent with a previous study’s findings [5] and validating our first hypothesis. Regarding medication expenditure, China has established a healthcare insurance system covering all citizens; however, the variations in mental health resources within different insurance schemes and geographical regions impact the reimbursement rates for schizophrenia medications. In China, the average reimbursement rate for antipsychotic medications is 46.2%, with a range of 44.9–69.4%. Thus, patients’ families bear a significant financial burden because of out-of-pocket medication expenses beyond the reimbursement proportion [32, 33]. In terms of adverse effects burden, some individuals might experience noticeable adverse reactions during antipsychotic medication usage; however, these effects disappear after discontinuation. Patients may experience a “worsening with treatment” phenomenon, and caregivers may encourage patients to reduce or discontinue medication because of inadequate understanding of the illness [34]. Therefore, psychiatric nurses should precisely identify the medication burden-related factors and reduce the medication burden in these patients to reduce adverse medication experiences

Our findings reveal that medication beliefs can directly predict medication experiences. Specifically, enhanced medication beliefs are associated with better medication experiences, which is consistent with a previous study’s results [17] and validates our second hypothesis. The Theory of Planned Behavior suggests that perceived value is the ratio of perceived benefits and risks [35]. In the context of schizophrenia requiring long-term antipsychotic medication, patients are more likely to develop positive medication beliefs and medication experiences when they establish good medication attitudes [36]. Therefore, psychiatric nurses should aim to increase patients’ beliefs in the necessity of medication while reducing their medication-associated concerns, thereby improving adverse medication experiences in individuals with schizophrenia

Our results demonstrate that medication beliefs play a partial mediating role in the relationship between medication burden and medication experience, thereby validating our third hypothesis. Medication beliefs are influenced by medication load in individuals with schizophrenia, consistent with previous research findings[18,19]. This can be explained by the fact that people with schizophrenia have a multifaceted medication burden, which leads to apprehensions regarding the treatment and subsequent adverse medication experiences. In shared decision-making, healthcare professionals and patients collaboratively choose appropriate treatment or care based on clinical evidence and patient preferences. Moreover, psychiatric nurses can form a collaborative alliance with physicians and patients to establish a healthcare team [37]. This alliance can help to assess the patient’s treatment preferences, analyze the medication burden during the intervention regime, participate in shared decision-making, and assist the patient in selecting treatment and rehabilitation plans with higher acceptability as well as minimal side effects. Consequently, these steps might enhance medication beliefs and contribute to positive medication experiences [38]

Our results have several implications for clinical nursing practice. Firstly, psychiatric nurses can improve their medication experiences by reducing the medication burden in individuals with schizophrenia. This involves promptly identifying adverse drug reactions, recognizing variations in pharmaceutical tolerance and efficacy, optimizing the risk/benefit ratios, correcting patient and caregiver cognitive biases, and enhancing family support systems. Leveraging the collaborative alliance between healthcare professionals and patients can also be effective for discussing treatment expenditure and shared decision-making. Hence, utilizing China’s existing mental health resources and policy benefits can reduce the medication burden for individuals with schizophrenia [39, 40]. Secondly, psychiatric nurses can enhance medication experiences by increasing medication beliefs in patients with heavy medication burdens. Thus, medication belief levels can be enhanced by providing health education regarding the etiology, symptoms, treatment regime, and prognosis as well as assisting such patients in understanding and accepting their illness. Additionally, psychiatric nurses can improve adverse medication experiences by facilitating patient engagement, exchanging information about treatment issues, exploring patient ambivalence, and assessing the benefits as well as drawbacks of antipsychotic medication treatment through shared decision-making [41, 42]

## Limitations

This study has some limitations that should be addressed. Firstly, our structural equation model was based on past studies and relevant theories. Since cross-sectional studies cannot infer causal relationships, between variables. longitudinal studies should be undertaken to validate our conclusions. Secondly, the study sample was limited to only two psychiatric specialty hospitals in China. Hence, multi-center studies should be conducted to confirm the generalizability of the research findings to other countries. Lastly, we focused on clarifying the underlying mechanisms of medication experiences through quantitative research. Since patients’ understanding of medication experiences is highly individualized, future research should adopt a mixed-methods approach. This approach could use quantitative data to understand the factors influencing medication experiences and concurrently collect qualitative data to summarize patients’ specific medication experiences. Hence, the integration of quantitative and qualitative research can enhance the mechanisms of action of relevant factors. This approach would also help in deducing the mechanisms by which medication experiences transpire in individuals with schizophrenia, determining the causes and importance of various involved influences

## Conclusion

Our findings indicate that medication burden directly impacts the medication experience of individuals with schizophrenia and medication belief plays a mediating role in the relationship between medication burden and experiences, respectively. Thus, psychiatric nurses can enhance the patients’ medication experience by reducing medication burden and strengthening medication beliefs. Therefore, the results also provide theoretical references and decision-making foundations for psychiatric nursing professionals to develop appropriate management strategies for individuals with schizophrenia

## Data Availability

Due to the privacy of the participants involved in the study data, the datasets generated and/or analyzed in the study are not currently publicly available but are available from the corresponding authors of this study upon reasonable request.
